# Supplementary material for: Effects of ionic strength on gating and permeation of TREK-2 K2P channels
Source: PLoS One. 2021 Oct 7;16(10):e0258275. doi: 10.1371/journal.pone.0258275 (PMC8496810; doi:10.1371/journal.pone.0258275)
Supplement: Attachment — Submitted filename: Response-to-reviewers.docx [file pone.0258275.s002.docx]

**[PONE-D-21-22346] Response to reviewers**

**Editor:**

**Based on my evaluation, I concur with the reviewer´s comments. However, I have three criticisms/suggestions that the I hope will help the authors to improve their work.**

**The unexpected finding that high concentrations of intracellular K decrease TREK K2P channel open probability by Tucker´s group is quite interesting. The authors propose that high ionic strength plays a role in this phenomenon; however, they also suggest a stably occupied filter by K underlies the reduced open probability caused by high K concentration. Thus, the authors need to make their conclusion more explicit to understand the role of ionic strength and selectivity filter occupation.**

We thank the editor for this question as it reveals a possible misunderstanding of the text. The evidence at hand is clearly not sufficient to link K^+^ occupancy with functional states. We merely want to express that the SF is most likely filled with K^+^ under high [K+] AND that it is predominantly closed (low Po) without implying any specific mechanistic causation in that statement. We have therefore replaced the relevant section with the following paragraph:

*“Stable, long closures were observed in high K^+^ concentrations in which the filter is likely to be stably occupied by K^+^ (Figs 3). Therefore it seems likely that the SF can be occupied by K^+^ and the channel simultaneously rendered non-conductive. This complements our knowledge of K^+^ depleted closed states of TREK that have emerged from functional* *(6) and structural studies* *(11).”*

**The data presented in Figure 4b shows that the conductance increased with the K concentration. However, I found the analysis less credible. The Michaelis-Menten equation used to analyse the conductance vs K concentration predicts that the conductance is 0 at 0 K. This does not seem to be the case. Also, the estimated Km (110 mM) is outside the K concentration range used in the experiments, making this Km value less realistic.**

Biophysical studies with [0] K on one side will be fraught with difficulty. As with the other extreme conditions deployed here it is highly likely that the protein structure could become disrupted. Selectivity filters in low K may become distorted, bound partially by Na and less conductive (Lolicato et al., 2020; Zhou and MacKinnon, 2004). Indeed it would therefore not be expected to provide an easy biochemical or quantitative description for these conditions and we agree that the MM equation does not hold in 0 M K^+^. We have deliberately not extended the dotted line of fits in the figure to this range and kept it to the data range sampled. The original manuscript already contains a qualifying comment and explanation about the quality and interpretation of the fits and we hope this is sufficient.

*“Fitting this relation to a Michaelis-Menten-model yields values of 343±40 pS (n > 4, patches) maximum conductance with a half maximal concentration of 110±59.8 mM for internal K^+^ (values for 80 mV, 95% confidence interval of fit estimates). This is in good agreement with similar measurements of the BK channel* *(29) although care should be taken with interpreting the K_m_ value here as the estimate lies at the bottom end of the K^+^ concentrations sampled and more accurate values of such parameters are typically obtained from measurements in symmetrical [K^+^] and in artificial bilayers* *(13,29).”*

We included this as we would like to highlight the important argument emerging from this figure i.e. that there is no large slope between 500 and 100 mM K in fig 3c, which is crucial for the choice of 1M KCl for the experiments done later on in fig 5.

**Finally, what is the explanation for the voltage-dependent effect of K on the conductance vs K concentration curves?**

That is clearly an enormous question that we would love to be able to answer properly from a molecular perspective. TREK-2 exhibits an asymmetric single-channel conductance, with outward currents being smaller than inward currents. This would suggest an asymmetric energy profile of the permeation pathway, resulting in different conditions for passage of K+ in two opposing directions. Additionally, inward and outward currents are also characterised by strikingly different gating kinetics. In a recent paper, we have modelled the voltage-dependent gating of single TREK-2 channels reconstituted in lipid bilayers [23]. We found that the complex voltage dependence of channel open and closed states can be described by two conformational changes of the channel gate that occur at membrane potentials above -20 and +60mV. Of the two conformational changes, the former (at -20mV) is responsible for different kinetics of inward and outward currents. Since TREK-2 channel gate is at the selectivity filter, this conformational change can be also expected to affect K+ permeation and thus single-channel conductance. The voltage-dependent effect of K+ on the conductance vs K+ concentration curves in Fig 4B may therefore be a combination of these two mechanisms but would not want to suggest that one is more important than the other at this stage.

**I suggest combining Figures 3 and 4 because they are closely related.**

Good idea – now done

**Reviewer #1:**

**This is a very convincing manuscript showing that TREK-2 is inhibited by the ionic strength of the internal medium. The control experiments are adequate. The authors also show that the effect of the opener BL149 on channel open probability is retained at high ionic strength but not the effect on channel unit conductance. This provides insight into how BL1249 modulates TREK-2. The data are well presented and discussed. The manuscript is very well written. We agree with the authors that the term "leak" for these channels is inappropriate when considering their electrophysiological properties, and that it would be better to return to the original description of background channels.**

***Lines 255-256: please replace Fig. 1C by Fig. 1D*
No other specific comments**

Done

R**eviewer #2:**

**In this study, Conrad et al investigating for the K+ dependent gating process of TREK-2 channel by using high concentrations of internal K+, found an inhibitory effect induced by the high K+ concentration of the recording solution. The authors present compelling evidence that this behavior is indeed dependent on the high ionic-strength of the internal solution. The authors also showed that the activator BL1249 was still able to increase the Po in high ionic-strength. I found this to be an interesting paper and observation that is well done, and I feel this will be of interest to the ion channel field. I have a few comments:

1. How to reconcile data from high ionic-strength and the activator BL1249? In discussion is proposed: “a poorly conductive but K+-filled filter conformation may also exist in TREK channels, and it is this conformation that presumably underlies the reduced Po in very high K+ concentrations.” However, BL1249 was still able to increase Po, any idea of the mechanism?**

This paragraph has been changed in response to the related question of the editor. We did not mean to suggest that K occupancy is causal to the channel closing, or incompatible with the channel opening. The likely mechanism for filter activation of BL1249 is via the SF backbone structure, but distinct from the binding site and mechanism of action of ML335.

The evidence here refines the mechanistic conclusions of Schewe et al. in as much as the conductance and K occupancy effects of BL1249 could be epiphenomena and that opening of the channel by BL1249 might occur through a conventional allosteric mechanism affecting the supporting network of the selectivity filter via the BL1249 binding site at its bottom. This mechanism appears to be unaffected by changes to ionic strength.

**2. There are some figures not properly indicated in the text, e.g., page 8, line 145 Fig. 3a should say Fig. 4a. Page 8, line 148, Fig. 3b should say 4b. Page 9, line 175, (See Fig. 5.1c) should say (see Fig. 1d) and Fig 1c should say Fig. 1d. Page 15, line 288, Fig. 3b should say 4b.**

All figure references have now been checked and updated after the merger of previous figs 3 and 4.
